# Novel *BRCA1* and *BRCA2* Tumor Test as Basis for Treatment Decisions and Referral for Genetic Counselling of Patients with Ovarian Carcinomas

**DOI:** 10.1002/humu.23137

**Published:** 2016-11-09

**Authors:** Robbert D.A. Weren, Arjen R. Mensenkamp, Michiel Simons, Astrid Eijkelenboom, Aisha S. Sie, Hicham Ouchene, Monique van Asseldonk, Encarna B. Gomez‐Garcia, Marinus J. Blok, Joanne A. de Hullu, Marcel R. Nelen, Alexander Hoischen, Johan Bulten, Bastiaan B.J. Tops, Nicoline Hoogerbrugge, Marjolijn J.L. Ligtenberg

**Affiliations:** ^1^ Department of Human GeneticsRadboud University Medical CenterNijmegenThe Netherlands; ^2^ Department of PathologyRadboud University Medical CenterNijmegenThe Netherlands; ^3^ Department of Clinical GeneticsGROW ‐ School for Oncology & Developmental BiologyMaastricht University Medical CentreMaastrichtThe Netherlands; ^4^ Department of Obstetrics & GynaecologyRadboud University Medical CenterNijmegenThe Netherlands

**Keywords:** ovarian cancer, *BRCA1*, *BRCA2*, cancer predisposition, *BRCA* testing, personalized medicine, PARP‐inhibitor, single molecule molecular inversion probes

## Abstract

With the recent introduction of Poly(ADP‐ribose) polymerase inhibitors, a promising novel therapy has become available for ovarian carcinoma (OC) patients with inactivating *BRCA1* or *BRCA2* mutations in their tumor. To select patients who may benefit from these treatments, assessment of the mutation status of *BRCA1* and *BRCA2* in the tumor is required. For reliable evaluation of germline and somatic mutations in these genes in DNA derived from formalin‐fixed, paraffin‐embedded (FFPE) tissue, we have developed a single‐molecule molecular inversion probe (smMIP)‐based targeted next‐generation sequencing (NGS) approach. Our smMIP‐based NGS approach provides analysis of both strands of the open reading frame of *BRCA1* and *BRCA2*, enabling the discrimination between real variants and formalin‐induced artefacts. The single molecule tag enables compilation of unique reads leading to a high analytical sensitivity and enabling assessment of the reliability of mutation‐negative results. Multiplex ligation‐dependent probe amplification (MLPA) and Methylation‐specific multiplex ligation‐dependent probe amplification (MS‐MLPA) were used to detect exon deletions of *BRCA1* and methylation of the *BRCA1* promoter, respectively. Here, we show that this combined approach allows the rapid and reliable detection of both germline and somatic aberrations affecting *BRCA1* and *BRCA2* in DNA derived from FFPE OCs, enabling improved hereditary cancer risk assessment and clinical treatment of ovarian cancer patients.

## Introduction

Ovarian carcinoma (OC) is one of the most frequently diagnosed types of cancer in females in Europe with an age‐standardized incidence rate of 13.1 per 100,000 [Ferlay et al., 2013]. OC is most frequently diagnosed in patients above the age of 65 years [Yancik, 1993; Lowe et al., 2013]. However, the average age of onset is lower in patients who carry an OC predisposing germline aberration [Prat et al., 2005; Weissman, et al., 2012]. During the last decades, a modest improvement in OC survival has been reported [Lowe et al., 2013], but due to the low mean age‐standardized 5‐year survival (37.6%), the estimated number of OC‐related deaths remains high in Europe (7.6 per 100,000; age‐standardized rates) [Ferlay et al., 2013; De Angelis et al., 2014].

A promising novel therapy for OC patients is based on the inhibition of poly(ADP‐ribose) polymerase (PARP), which is synthetically lethal in cancer cells with acquired inactivation of the homologous recombination‐mediated repair pathway [Bryant et al., 2005; Farmer et al., 2005]. Multiple clinical trials with PARP inhibitors, including olaparib and niraparib, have demonstrated tolerability and efficacy of these treatments in OC patients [Audeh et al., 2010; Sandhu et al., 2013]. Moreover, progression‐free survival of OC patients is further improved when olaparib is administered in combination with other treatments (e.g., paclitaxel, carboplatin, and cediranib) [Liu et al., 2014; Oza et al., 2015]. Since PARP inhibitors are predominantly lethal for cells that have lost the ability of homologous recombination‐mediated repair, patients who have developed tumors with defects in this pathway show the highest response rates to such treatment [Mateo et al., 2015]. The highest response rates to treatments with olaparib were observed in OC patients with mutations affecting the homologous recombination genes *BRCA1* (MIM# 113705) or *BRCA2* (MIM# 600185) [Audeh et al., 2010; Ledermann et al., 2014]. Since genomic aberrations affecting *BRCA1* and *BRCA2* are among the most prevalent mutations observed in OCs [Cancer Genome Atlas Research, 2011; Kanchi et al., 2014; Patch et al., 2015], a substantial number of OC patients may benefit from treatments with PARP inhibitors.

Genomic aberrations affecting *BRCA1* and *BRCA2* are frequently encountered in both sporadic and familial OCs [Cancer Genome Atlas Research, 2011; Kanchi et al., 2014] (OMIM #604370 and #612555). Approximately 10%–15% of all OC patients carry a pathogenic germline aberration in *BRCA1* or *BRCA2* [Daly et al., 2010; Hennessy et al., 2010; Kanchi et al., 2014]. Loss of heterozygosity (LOH) of the wild‐type allele is the tumor‐initiating second hit in the majority of these patients [Foster et al., 1996; Berchuck et al., 1998]. Somatic mutations in *BRCA1* and *BRCA2* are observed in approximately 3.5%–8.5% and 2.5%–4% of OCs without an underlying germline mutation, respectively [Merajver et al., 1995; Foster et al., 1996; Berchuck et al., 1998; Cancer Genome Atlas Research, 2011; Kanchi et al., 2014]. Hypermethylation of the promoter of *BRCA1* is observed in approximately 10%–15% of these carcinomas [Baldwin et al., 2000; Bianco et al., 2000; Esteller et al., 2000; Cancer Genome Atlas Research, 2011]. Importantly, germline mutations, somatic mutations, and promoter hypermethylation appear mutually exclusive in OCs [Cancer Genome Atlas Research, 2011; Dworkin et al., 2009]. In total, *BRCA1* and *BRCA2* are mutated in 19%–22% of OCs and, consequently, these patients may benefit from PARP‐inhibitor treatment [Hennessy et al., 2010; Cancer Genome Atlas Research, 2011; Kanchi et al., 2014]. Based on the genetic heterogeneity of the observed mutation spectrum, sequencing of the entire open‐reading frame (ORF) of *BRCA1* and *BRCA2* using tumor‐derived DNA is required to identify the patients who may benefit from this treatment.

Sequencing of *BRCA1* and *BRCA2* using tumor‐derived DNA is hampered by the complexity of these genes, the low quality of the DNA derived from formalin‐fixed, paraffin‐embedded (FFPE) tumor samples and the low percentage of neoplastic cells in these samples. Several next‐generation sequencing (NGS) approaches to determine the mutation status of *BRCA1* and *BRCA2* have been developed, but most approaches were validated using high‐quality DNA (i.e., blood‐derived DNA) [Feliubadalo et al., 2013; Hirotsu et al., 2015; Strom et al., 2015]. Therefore, these approaches can successfully be implemented in a diagnostic setting to screen for germline defects in *BRCA1* and *BRCA2* using blood‐derived DNA [D'Argenio et al., 2015; Trujillano et al., 2015], but cannot be used to sequence low quality and highly fragmented DNA derived from FFPE tumor blocks. Recently, three multiplex PCR‐based targeted NGS methods to sequence *BRCA1* and *BRCA2* in DNA derived from FFPE material have been evaluated [Ellison, et al., 2015; Mafficini, et al., 2016]. However, these methods have relatively low levels of amplicon tiling, do not allow for strand‐specific amplification, and lack single molecule tagging. As a consequence, possible drop‐outs of amplicons and PCR jackpotting effects may result in false‐negative results [Ellison et al., 2015] or false‐positive calls due to deamination artefacts [Lou et al., 2013; Chen et al., 2014; Wong et al., 2014] and detection of low frequency variants is hampered [Jabara et al., 2011; Hiatt et al., 2013].

Here, we show that single‐molecule molecular inversion probe (smMIP)‐based targeted sequencing [Hiatt et al., 2013] is a reliable method to detect both germline and somatic mutations in *BRCA1* and *BRCA2* in FFPE samples, which can be applied to identify OC patients who may benefit from treatments with PARP inhibitors and are at high risk of carrying a germline *BRCA* mutation.

## Materials and Methods

### Patient Selection

A retrospective cohort of OC patients who were tested for germline *BRCA1* and *BRCA2* mutations after genetic counselling at the department of Human Genetics of the Radboudumc or the MaastrichtUMC+ was selected. All patients included signed informed consent for the use of stored material for research purposes (Radboudumc) or did not refuse use of stored material for research purposes (according to local policy, MaastrichtUMC+). OC derived from patients with a pathogenic germline *BRCA1* or *BRCA2* mutation were included in our study regardless of the histological phenotype. In contrast, of patients without a germline mutation only those reported as serous OC were selected. Histological revision was performed for all tumors by an expert pathologist (MS and JB). FFPE OCs were obtained between 1998 and 2014 at different time points during treatment and were categorized as either diagnostic (biopsy or primary debulking operation) or postchemotherapy (interval or secondary debulking operation). This study was approved by the medical ethics committee/institutional board (CMO, study 2014‐1472) of the Radboudumc.

### Isolation and Quality Assessment of FFPE Ovarian Cancer‐Derived DNA

DNA isolation from FFPE OC samples was performed using standard procedures. First, 200 μl of 5% Chelex‐100, diluted in TET lyses buffer with GlycoBlue, and 20 μl proteinase K (20 mg/ml) were added to the isolated sections of the FFPE ovarian cancer. This sample was sequentially incubated and mixed (350 rpm) at 56°C for 16hr, at 37°C for 48hr, and 95°C for 10 min. Incubation was followed by centrifugation at 16,000*g* for 1 min at room temperature and the supernatant was collected. Next, 20μl of NaAc (3M, pH 5.2) and 440μl ice‐cold ethanol (EtOH) was added to the supernatant (on ice) and mixed. After centrifugation at 16,000*g* for 10 min at 4°C, the supernatant was removed and the remaining pallet was washed with 1 ml ice‐cold 70% EtOH. To remove the EtOH, the sample was centrifuged at 16,000*g* for 2 min at 4°C and the supernatant was removed. The pellet was air‐dried and, subsequently, dissolved in 80μl of TE and incubated for 5 min at 56°C. The DNA concentration was determined (Qubit Fluorometer; Life Technologies, Carlsbad, CA) and amplifiability of the DNA was assessed by PCR‐based amplification of DNA fragments of 115 and 216 bp (see Supp. Data). Failed amplification of DNA fragments of 115 and 216 bp would probably inhibit subsequent proper smMIP‐based targeting of the regions of interest since DNA fragments of 152bp are captured using this approach.

### Targeted Sequencing of *BRCA1* and *BRCA2* by smMIPs

A total of 157 and 260 smMIPs were designed, covering all coding regions and intron–exon boundaries of *BRCA1* and *BRCA2*, according to previously published methods [O'Roak et al., 2012; Hiatt et al., 2013; Boyle et al., 2014] with minor modifications (see Neveling et al., in press; and Supp. Table S1). Briefly, these 75–80 bp long oligonucleotides (i.e., smMIPs; ordered from Integrated DNA Technologies, Interleuvenlaan, Belgium) contained extension and ligation arms (40‐45bp) and a linker sequence (30bp) and were designed to capture a target region of 112 nucleotides. In addition, all smMIPs contained a stretch of five random nucleotides (molecular tag), enabling the detection of 1,024 unique (tagged) reads per smMIP. Both DNA strands (i.e., plus and minus strand) of the target regions were targeted by independent smMIPs, enabling double tiling of these regions of interest. If the extension or ligation arm targeted a common SNP (MAF > 1%), two different smMIPs were designed to recognize and target both alleles. Next, smMIPs were pooled in an equimolar manner and phosphorylated by adding T4 polynucleotide kinase and 10x T4 DNA ligase buffer supplied with 10mM ATP (New England Biolabs, Ipswich, MA). To improve proper and equal coverage of the target regions, the smMIP pool was rebalanced based on initial sequencing results obtained using reference (blood‐derived) DNA [Neveling et al., in press]. Targeted sequencing of *BRCA1* and *BRCA2* using DNA derived from FFPE OCs was performed as previously described [Weren et al., 2015], using a slightly modified smMIP capture protocol [O'Roak et al., 2012; Hiatt et al., 2013]. Briefly, smMIP capture was performed on 10μl of input DNA (20–500ng) supplied with 15μl capture mixture (0.01μl ampligase DNA ligase [100U/μl; Illumina, Madison, WI], 2.5μl 10x ampligase buffer [Illumina], 0.27μl smMIP pool dilution [6.6x10^5^ μM], 0.32μl Hemo Klentaq [10U/μl; New England Biolabs], 0.03μl dNTPs [0.25mM], and 11.9μl H_2_O). The mixture was incubated at 95°C for 10 min, and subsequently at 60°C for 24 hr. Incubation was followed by exonuclease treatment: 0.5μl exonuclease I (New England Biolabs), 0.5μl exonuclease III (New England Biolabs), 0.2μl 10x ampligase buffer (Illumina), and 0.8μl H_2_O was added to the (cooled) capture samples (consecutively incubated at 37°C and 95°C for 45 and 2 min, respectively). Subsequently, 10μl of the sample was used to perform a PCR reaction by adding 1.3μl of barcoded reverse primer (10μM; Illumina), 12.5μl 2x iProof (BioRad Laboratories, Veenendaal, the Netherlands), 0.125μl forward primer (100μM; Illumina), and 1.075μl H_2_O (final volume: 25μl; PCR program: 98°C, 30 sec – 24x [98°C, 10 sec ‐ 60°C, 30 sec ‐ 72°C, 30 sec] ‐ 72°C, 2 min ‐ 4°C, *∞*). Sequencing of the barcoded samples was performed using the Illumina NextSeq500 system, with 2 × 151‐bp paired‐end reads; smMIP libraries required spike‐in of custom primers as described previously [O'Roak et al., 2012]. On average, 44 OC samples were sequenced per NextSeq500 Mid Output run. Obtained bcl files were converted into fastq files that were separated by barcode. Double tiling was achieved for up to 99.3% of the ORF of *BRCA1* and *BRCA2*, including the –20 and +20 intronic regions.

Fastq files were analyzed using the SeqNext software package (version 4.2.2; JSI Medical Systems GmbH, Kippenheim, Germany). Briefly, based on the single‐molecule tag, consensus reads were generated and variants in coding regions were called if present in ≥ 5% of all reads and ≥3 unique variant reads. For details, see Supplementary Data.

### Sequencing Data Analysis

Fastq files were analyzed using the SeqNext software package (version 4.2.2; JSI Medical Systems GmbH, Kippenheim, Germany). First, sequencing read data (fastq files) were mapped to exonic regions plus adjacent intronic regions of *BRCA1* (NM_007294.3) and *BRCA2* (NM_000059.3). Reads with undefined nucleotides in their barcodes or of low quality were ignored, and to generate consensus reads, all bases should be sequenced at least once in the consensus reads, at least two tagged reads are required to create a consensus read, and reads with less than 30% consensus were discarded. Reads were excluded from alignment if these contain more than 15% mismatches compared with the reference or if less than 50% of the bases match to the reference. The minimal absolute sequencing depth and expected sequencing depth for variant calling were 20 and 30 unique reads, respectively. In addition, variants were called if the variant was observed in ≥ 5% of all reads and ≥ 3 unique variant reads were present. To exclude (FFPE induced) cytosine deamination artefacts, C:G>T:A transition calls were manually discarded if the variant was not present in the smMIP covering the opposite strand (i.e., targeting the guanine nucleotide). Subsequently, all variants in coding regions and the intron–exon boundaries of *BRCA1* and *BRCA2* were selected for analysis. Variants were considered common SNPs if these were observed in our in‐house database of *BRCA1* and *BRCA2* germline variants (accessed 01/03/2016; our in‐house database contains 264 and 413 germline variants (in the close proximity) of the *BRCA1* and *BRCA2* locus, respectively). We previously applied *BRCA* sequencing (i.e., exonic‐ and adjacent intronic regions) on blood‐derived DNA from 76 patients (of whom 90 of the 107 OCs were derived) using Sanger‐ or Iontorrent‐based sequencing prior to this study. All germline variants in *BRCA1* and *BRCA2* that were identified in these screenings were included in our analysis to confirm that these variants were also present in the smMIP‐based targeted sequencing data derived from the patient‐matched FFPE samples. The other 17 patients had only been evaluated for the mutation segregating in their family. Insertion and deletion calls in regions known to be prone for false‐positive calls (i.e., variant calls in homopolymer stretches of eight adenine residues and variant calls in nucleotides at the end of a sequencing read, which were not present in the sequencing reads derived from overlapping smMIPs) were considered false positives. Remaining variants were validated using Sanger sequencing or by independent resequencing of the corresponding sample using our smMIP‐based targeted sequencing approach. Variants were submitted to the locus‐specific databases at LOVD: www.lovd.nl/BRCA1, www.lovd.nl/BRCA2.

To determine the accuracy per nucleotide of our method, we determined the number of true positives (*n* = 996), false positives (*n* = 14), and false negatives (*n* = 18) based on the results of 107 samples. The number of true negatives (*n* = 1,442,122) was determined based on 90 samples of which both *BRCA* genes were completely sequenced using Sanger‐ or Iontorrent‐based sequencing of blood‐derived DNA prior to the smMIP‐based NGS analysis.

### Multiplex Ligation‐Dependent Probe Amplification Methylation‐Specific Multiplex Ligation‐Dependent Probe Amplification

Multiplex ligation‐dependent probe amplification (MLPA) was performed according to the manufacturers protocol to detect intragenic copy‐number variations affecting *BRCA1* [kit P077; MRC Holland, Amsterdam, The Netherlands]. For data analysis, the GeneMarker software (Softgenetics, State College, PA) was used using the population normalization mode. Using this population normalization mode, possible chromosomal aberrations at other genomic regions (e.g., aneuploidies) would not hamper the MLPA analysis (in contrast to other approaches that only use probes outside the genomic locus of *BRCA1* as a reference). In short, MLPA analysis was performed with 38 probes targeting the *BRCA1* locus and 10 probes targeting other chromosomes. For data analysis, peak intensities were adjusted based on the average of peak intensities from all probes (i.e., population normalization). Methylation‐specific multiplex ligation‐dependent probe amplification (MS‐MLPA) was performed and analyzed according to the manufacturers protocol (kit ME001 C2) (MRC Holland).

### LOH Analysis

LOH of the *BRCA1* and the *BRCA2* locus was determined using the variant allele frequency (VAF) of common SNPs (*n* = 16 for *BRCA1*; *n* = 20 for *BRCA2*) and confirmed germline variants called in a heterozygous state (5%≤ VAF≤95%). As 95% of the samples had a neoplastic cell percentage of at least 32% (median 65%, range 15%–90%), an average major VAF of >60% was considered as a marker for LOH of the corresponding locus. Noteworthy, LOH analysis may have been hampered by the low percentage of neoplastic cells in a minor subset of these samples.

## Statistics

A two‐tailed Fisher exact test was applied to determine whether the frequency of high‐grade serous OCs statistically differed between patients with a germline mutation in *BRCA1* or *BRCA2*. The predetermined level of significance was *P* = 0.05.

## Results

### Sample Selection and Coverage of smMIP‐Based Sequencing of *BRCA1* and *BRCA2*

**Figure 1:**
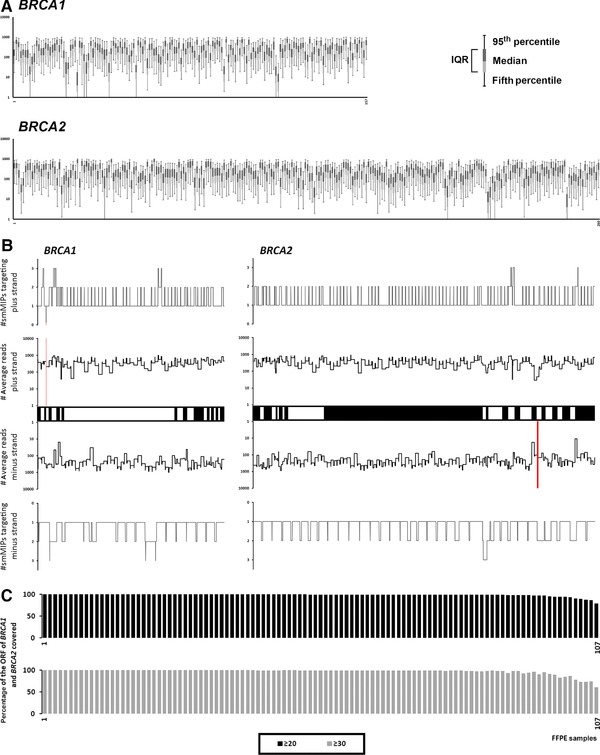
**A**: Average number of unique reads per smMIP. X‐axis: number of unique smMIPs included in our panel to sequence *BRCA1* (*n* = 157 smMIPs) and *BRCA2* (*n* = 260 smMIPs) (ordered for the genomic position of the target). Y‐axis: number of unique reads obtained per smMIP. The average and median number of unique reads per smMIP is 254 and 154, respectively. Numbers are based on sequencing results obtained from 107 FFPE samples (for details; see main text or *Materials and Methods*). **B**: Number of unique reads/smMIPs mapping to/targeting the plus or minus strand of the ORF of *BRCA1* and *BRCA2*. smMIPs targeting plus/minus strand: total number of smMIPs targeting the plus/minus strand of the corresponding base‐pair position (range 0–3 smMIPs per strand per position). Average reads plus/minus strand: average number of unique reads mapping to the plus and minus strand based on 107 ovarian carcinoma samples. On average, the plus/minus strand were covered 356x/291x (*BRCA1*) and 334x/258x (*BRCA2*), respectively. Bars on the X‐axis represent the nucleotides located in the exons of *BRCA1* and *BRCA2*, including the canonical splice sites. Red bars indicate regions (4 and 16 bp, respectively) without any smMIPs or mapped reads at the corresponding locus. Note that 100% of the ORF including the canonical splice sites is sequenced and that 99.8% is sequenced on both the plus and minus strand. **C**: Percentage of the ORF of *BRCA1* and *BRCA2* covered with at least 20 and 30 unique reads in 107 FFPE ovarian carcinoma samples. On average, 98.8% (median 99.8%, range 79.3%–100%) and 97.4% (median 99.6, range 60.9%–100%) of the ORF of *BRCA1* and *BRCA2*, including the −20 and +20 intronic regions, was covered with a sequencing depth of at least 20x and 30x, respectively.

For the evaluation of our approach, 127 ovarian tumor samples derived from 96 patients were tested: 29 with a *BRCA1*, 14 with a *BRCA2*, and 53 without a germline mutation in either gene. For 20 samples, including 16 samples that were poorly amplifiable based on our control PCRs, the sequencing depth of the ORF of *BRCA1* and *BRCA2* and total number of mapped reads was low (Supp. Fig. S1A and B). In the remaining 107 samples, the average number of unique reads per smMIP (after deconvolution of the PCR duplicates) was high (Fig. 1A). The average number of unique reads per coding base pair (including canonical splice sites) was 647 for *BRCA1* (146–1,476) and 592 for *BRCA2* (47–1,679) (Supp. Fig. S1C) (for the total number of tagged reads, see Supp. Fig. S1D). These unique reads were equally mapped to the plus and minus strands of the ORF, revealing that both strands were successfully targeted by our method (Fig. 1B). On average, 98.8% and 97.4% of the ORFs including the −20 and +20 intronic regions of *BRCA1* and *BRCA2* were covered with at least 20 and 30 unique reads reflecting a 95% chance of detecting a variant with a VAF of 30% and 20%, respectively (Fig. 1C).

### Interpretation of smMIP‐Based Mutation Detection

**Figure 2:**
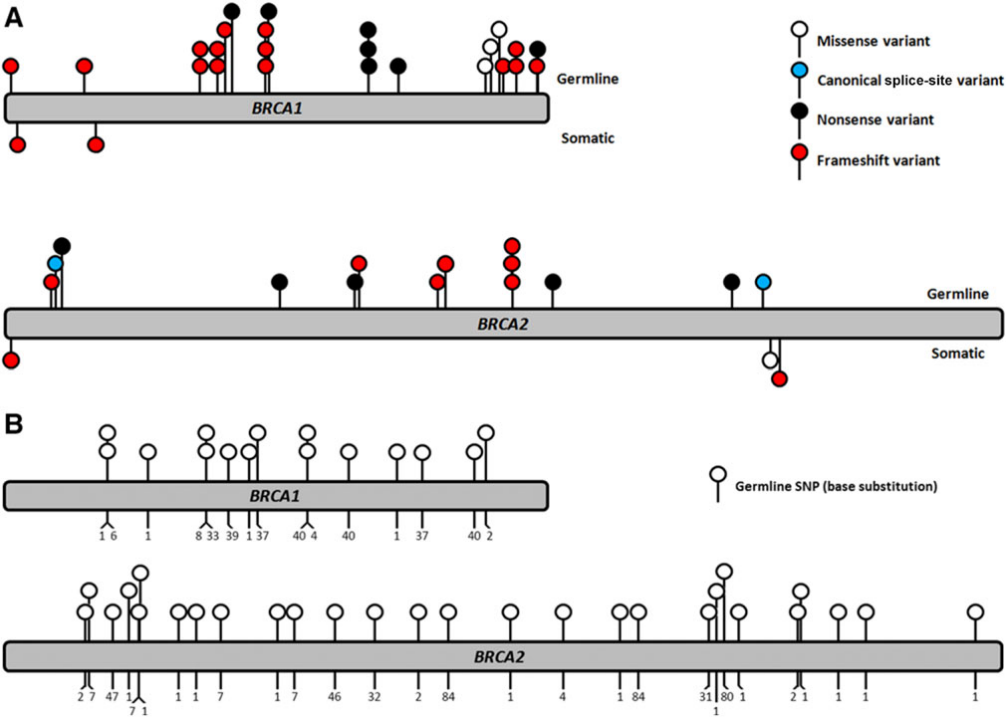
**A**: Pathogenic germline and somatic mutations in *BRCA1* and *BRCA2* detected using smMIP‐based targeted sequencing of FFPE tumor material. Lollipops above the bar: germline mutations detected in 47 FFPE ovarian carcinomas derived from 38 patients. Lollipops below the bar: somatic pathogenic mutations observed in seven FFPE ovarian carcinomas derived from five patients. **B**: Genomic location of 43 germline SNPs in *BRCA1* and *BRCA2* that were selected to determine the sensitivity of smMIP‐based next‐generation sequencing. Depicted base substitutions (lollipops) represent 15 and 28 benign germline variants in the ORF of *BRCA1* and *BRCA2*, respectively. These variants were known to be present in the germline of a subset of the included ovarian carcinoma patients prior to smMIP‐based sequencing of *BRCA1* and *BRCA2* in the corresponding FFPE ovarian carcinomas. Numbers depict the total number of the corresponding germline variant observed in these ovarian carcinoma patients. All germline SNPs could successfully be detected using smMIP‐based targeted sequencing on DNA derived from the corresponding FFPE ovarian carcinoma sample.

All pathogenic germline mutations in *BRCA1* (*n* = 31) and *BRCA2* (*n* = 16), known prior to smMIP‐based targeted sequencing, were called by the NextSeq software in 47 tumor samples derived from 38 patients (Fig. 2A). The average number of unique variant reads and percentage of variant calls was high for these variants in both *BRCA1* (524 [28–2,125]; 82.3% [64%–95%]) and *BRCA2* (686 [23–2,832;, 71.3% [45%–94%]) (Table 1). Moreover, 745 out of 763 class 1/2 germline variants, known prior to smMIP‐based targeted sequencing in 90 samples, were called (Fig. 2B). Based on the detection of 792/810 germline variants, an overall sensitivity of 97.8% (95% CI, 96.8%–98.8%) is estimated. The positions of the 18 variants that were not called using our standard variant calling settings were inspected visually. Five variants were missed due to LOH leading to a low percentage of variant reads in samples with a high tumor cell percentage. Thirteen variants were false negative due to a poor read depth at four SNP positions in five samples with a low number of total mapped reads (< 20,000 unique reads) (Supp. Fig. S1E).

**Table 1:** Variant Calling of Germline Mutations in *BRCA1* and *BRCA2* in FFPE Samples

Patient ID	Germline mutation	Nucl. change	AA change	Carcinoma phenotype	^#^FFPE samples	Neoplastic cells (%)	#Var. reads	Var. reads (%)	LOH
P040	*BRCA1*	c.3748G>T	p.Glu1250^*^	High‐grade serous	3	70	127	64	Y
						30	352	75	Y
						80	709	80	Y
P016	*BRCA1*	c.5266dup	p.Gln1756fs	High‐grade serous	2	70	77	80	Y
						60	756	80	Y
P038	*BRCA1*	c.4964C> T	p.Ser1655Phe	High‐grade serous	2	60–70	58	87	Y
						70	97	74	Y
P057	*BRCA1*	c.5137delG	p.Val1713fs	High‐grade serous	2	40	993	68	Y
						90	1,120	82	Y
P070	*BRCA1*	c.3748G>T	p.Glu1250^*^	High‐grade serous	2	65	102	79	Y
						50–60	411	76	Y
P078	*BRCA1*	c.5266dup	p.Gln1756fs	Clear cell	2	40	2,125	79	Y
						65	215	79	Y
P001	*BRCA1*	c.3748G>T	p.Glu1250^*^	Poorly/undifferentiated	1	50	58	87	Y
P007	*BRCA1*	c.2685_2686del	p.Pro897fs	High‐grade serous	1	90	57	95	Y
P022	*BRCA1*	c.5485dup	p.Glu1829fs	High‐grade serous	1	90	405	90	Y
P025	*BRCA1*	c.2019del	p.Glu673fs	High‐grade serous	1	90	28	93	Y^a^
P029	*BRCA1*	c.68_69del	p.Glu23fs	High‐grade serous	1	85	150	83	Y
P035	*BRCA1*	c.2722 G>T	p.Glu908^*^	High‐grade serous	1	75	358	88	Y
P036	*BRCA1*	c.2197_2201del	p.Glu733fs	High‐grade serous	1	80	483	89	Y
P039	*BRCA1*	c.5095C>T	p.Arg1669Trp	High‐grade serous	1	70	322	84	Y
P046	*BRCA1*	c.2338C>T	p.Gln780^*^	High‐grade serous	1	90	1,100	91	Y
P048	*BRCA1*	c.815_824dup	p.Thr276fs	High‐grade serous	1	60–70	150	68	Y
P066	*BRCA1*	c.2269delG	p.Val757fs	High‐grade serous	1	80–90	667	88	Y
P076	*BRCA1*	c.2685_2686del	p.Pro897fs	Mixed carcinoma	1	65	1,602	88	Y
P077	*BRCA1*	c.4057G> T	p.Glu1351^*^	High‐grade serous	1	80	751	92	Y
P079	*BRCA1*	c.2019del	p.Glu673fs	Poorly/undifferentiated	1	90	190	86	Y
P085	*BRCA1*	c.2197_2201del	p.Glu733fs	Mixed carcinoma	1	70	1,134	90	Y
P093	*BRCA1*	c.5095C>T	p.Arg1699Trp	High‐grade serous	1	65	265	80	Y
P094	*BRCA1*	c.2685_2686del	p.Pro897fs	High‐grade serous	1	85	590	88	Y
P096	*BRCA1*	c.5503C>T	p.Arg1835^*^	High‐grade endometrioid	1	60	786	68	Y
P028	*BRCA2*	c.4449del	p.Asp1484fs	High‐grade serous	2	40	23	52	Y
						30	282	63	Y
P086	*BRCA2*	c.3639_3652del	p.Val1214fs	High‐grade serous	2	35	172	64	P^b^
						10–50	524	56	P^b^
P006	*BRCA2*	c.4533del	p.Glu1511fs	High‐grade serous	1	65	90	85	Y
P021	*BRCA2*	c.3599_3600del	p.Cys1200^*^	High‐grade serous	1	50	138	55	Y
P034	*BRCA2*	c.2830A>T	p.Lys944^*^	High‐grade serous	1	70	727	92	Y
P044	*BRCA2*	c.516+1G>T	p.?	High‐grade serous	1	70	409	52	N
P067	*BRCA2*	c.582G>A	p.Trp194^*^	High‐grade serous	1	45	2,832	84	Y
P068	*BRCA2*	c.5645C> G	p.Ser1882^*^	High‐grade serous	1	60	89	79	Y
P069	*BRCA2*	c. 469_470del	p.Lys157fs	High‐grade serous	1	40	515	45	N
P071	*BRCA2*	c.5213_5216del	p.Thr1738fs	High‐grade serous	1	80	1,351	89	Y
P072	*BRCA2*	c.5213_5216del	p.Thr1738fs	High‐grade serous	1	55	1,299	90	Y^a^
P073	*BRCA2*	c.7480C> T	p.Arg2494^*^	High‐grade serous	1	50–90	81	66	Y
P074	*BRCA2*	c.7806‐1G>T	p.?	High‐grade serous	1	55	1,207	75	Y
P075	*BRCA2*	c.5213_5216del	p.Thr1738fs	Poorly/undifferentiated	1	90	1,232	94	Y

The indicated nucleotide change is based on the cDNA sequence.

a Based on pathogenic germline variant call in FFPE material; no additional informative SNPs available.

b Possibly LOH, but the analysis is hampered by the low percentage of tumor cells in the corresponding FFPE sample.

1,135 variants were called in 107 samples. In addition to the 47 pathogenic germline mutations, 937 of these variants were known benign germline variants (class 1 or 2) present in our in‐house database of *BRCA1* and *BRCA2* variants. Of the remaining 151 variants, 125 were C:G to T:A transitions reflecting FFPE‐induced cytosine deamination artefacts (Supp. Table S2). The majority of these (*n* = 64) were observed in only two samples. In total, 12 variants were somatic mutations as they were confirmed present in the tumor, but absent in germline DNA. Five different somatic mutations, represented in eight carcinomas from five patients, were considered pathogenic, whereas the other four somatic variants were considered benign passenger mutations (Table 2). The remaining 14 variants were false‐positive variant calls due to sequencing artefacts. They were called with a low percentage and number of variant reads (Supp. Fig. S2A and B) at either the last nucleotide of the targeted region or in a stretch of eight adenosines. Thus, after exclusion of deamination artefacts, the percentage of false‐positive calls is low (1.4%, 14/1,010).

**Table 2:** Variant Calling of Somatic Mutations in *BRCA1* and *BRCA2* in FFPE Samples

Patient ID	Gene	Nucleotide change	Amino acid change	Carcinoma phenotype	^#^FFPE samples	Neoplastic cells (%)	^#^Var. reads	Var. reads (%)	Pathogenic^a^	LOH
P047	*BRCA1*	c.121del	p.His41fs	High‐grade serous	2	60–70	486	41	Yes	Yes
						35	53	16		P^b^
P061	*BRCA1*	c.929del	p.Gln310fs	High‐grade serous	2	60	18	24	Yes	Yes
						50	19	23		Yes
P050	*BRCA2*	c.7971dup	p.Tyr2658fs	Mixed carcinoma	2	50	689	52	Yes	V
						Yes	634	66		Yes
P062	*BRCA2*	c.51_52del	p.Arg18Leufs	Poorly/undifferentiated	1	90	789	75	Yes	Yes
P091	*BRCA2*	c.7878G>C	p.Trp2626Cys	High‐grade serous	1	70	339	47	Yes	Yes
P048^c^	*BRCA2*	c.6970C>G	p.His2324Asp	High‐grade serous	1	60–70	235	37	No^d^	No
P063	*BRCA2*	c.4154C>T	p.Ser1385Leu	Low‐grade endometrioid	1	30	55	8	No^e^	Yes
P063	*BRCA2*	c.4347C>G	p.Phe1449Leu	Low‐grade endometrioid	1	30	128	6	No^f^	Yes
P079^c^	*BRCA2*	c.8599A>C	p.Thr2867Pro	Poorly/undifferentiated	1	90	59	36	No^g^	No

The indicated nucleotide change is based on the cDNA sequence.

a Variants were considered pathogenic as they were either truncating or a known class 5 missense mutation and considered benign passenger mutations based in in silico prediction tools.

b Probably LOH, but the analysis is hampered by the low percentage of tumor cells in the corresponding FFPE sample.

c Patients with a *BRCA1* germline mutation.

d Weakly conserved nucleotide (PhyloP: 1.90), Align GVGD: class C0 (GV: 130.59; GD: 22.66), SIFT: tolerated (score: 0.39).

e Weakly conserved nucleotide (PhyloP: 1.58), Align GVGD: class C0 (GV: 353.86 ‐ GD: 0.00), SIFT: tolerated (score: 0.51).

f Weakly conserved nucleotide (PhyloP: 0.29), Align GVGD: class C0 (GV: 180.03 ‐ GD: 0.00), SIFT: tolerated (score: 1).

g Weakly conserved nucleotide (PhyloP: 0.61), Align GVGD: class C0 (GV: 129.31 ‐ GD: 1.62), SIFT: tolerated (score: 0.07).

For more details regarding these in silico predictions, see Supplementary Data.

In total, 16,033 nucleotides were analyzed per sample. Therefore, based on these results, the accuracy per nucleotide of our method is 99.998% (996 true positive, 14 false positive, 1,442,122 true negative, and 18 false negative).

### Detection of Copy‐Number Variants Affecting *BRCA1*

All 127 DNA samples were screened for copy‐number variants (CNVs) affecting the genomic locus of *BRCA1* using MLPA. Prior to this screening, it was established that three of these patients carried a germline deletion encompassing exon 22 of *BRCA1*. These deletions were confirmed in all FFPE OC samples (*n* = 4) derived from these patients. No CNVs were detected in the other FFPE OC samples. Noteworthy, the relative number of unique reads covering exon 22 was strongly decreased in the smMIP NGS data derived from patients with a germline deletion affecting this exon (Supp. Fig. S3).

### Detection of *BRCA1* Promoter Methylation

Possible methylation of the *BRCA1* promoter was determined using a MS‐MLPA assay for all 127 DNA samples. Methylation of the promoter of *BRCA1* was not observed in FFPE OC samples derived from patients with a germline or somatic pathogenic mutation affecting *BRCA1* (*n* = 43) or *BRCA2* (*n* = 22). In contrast, methylation of the *BRCA1* promoter was observed in 13 samples derived from 17% of the sporadic patients (9/53). In all 24 patients, from whom multiple OC specimen were available, the methylation of the *BRCA1* promoter was fully concordant in tumor samples at diagnosis and at interval or secondary debulking (Supp. Table S3).

### LOH

LOH was based on the allele frequency of *BRCA1* and *BRCA2* mutations and germline variants (*n* = 31 for *BRCA1*; *n* = 49 for *BRCA2*) that were called in a heterozygous state (5%≤ VAF≤ 95%) (Supp. Fig. S4). Informative SNPs in *BRCA1* and *BRCA2* were observed in 100 and 103 samples, respectively (Supp. Table S4). In concordance with *BRCA1* germline mutant allele frequencies of > 60% that suggest loss of the wild‐type allele (Table 1), all FFPE samples derived from patients with a pathogenic germline mutation in *BRCA1* revealed LOH of the *BRCA1* locus. Similarly, LOH is presumed in the tumor samples from the two sporadic patients with a somatic pathogenic *BRCA1* mutation and in all samples with *BRCA1* promoter methylation (Supp. Table S5).

LOH at the *BRCA2* locus was observed in 73% of the FFPE samples derived from carriers of a pathogenic germline mutation in *BRCA2* (Supp. Table S4). Based on these data, we consider that LOH occurred in the OC of 12 out of 14 patients (86%) with a *BRCA2* germline mutation (Table 1). LOH of the wild‐type allele is presumed in all four lesions with a somatic pathogenic *BRCA2* mutation (Table 2; Supp. Table S5).

LOH of the respective loci is not an indication for a *BRCA1* or *BRCA2* mutation. LOH at the *BRCA1* locus was observed in 73% of the tumors with a pathogenic germline mutation in *BRCA2* and in 80% of the tumors without a somatic pathogenic mutation in *BRCA1* or methylation of the *BRCA1* promoter. LOH of the *BRCA2* locus was observed in 56% of tumors with a pathogenic germline mutation in *BRCA1* and in 46% of the sporadic tumors without a somatic pathogenic mutation in *BRCA2* (Supp. Tables S4 and S5).

### Histology Review of the OCs

Histology revision by an expert pathologist revealed that 83% of the carcinomas derived from germline mutation carriers had a high‐grade serous histology (*n* = 35). The other patients with a germline mutation in *BRCA1* or *BRCA2* presented carcinomas with a mixed (*n* = 2), high‐grade endometrioid (*n* = 1), clear cell (*n* = 1), and poorly/undifferentiated (*n* = 3) histology (Supp. Table S6A). The frequency of high‐grade serous OCs did not differ between carcinomas derived from patients with a germline mutation in *BRCA1* (22/28) or *BRCA2* (13/14) (*P* = 0.39).

Although OCs of patients without a germline *BRCA1* or *BRCA2* mutation were selected based on their reported serous histology, revision revealed that 13% of these patients did not develop low‐ or high‐grade serous carcinomas (seven out of 54). These patients developed carcinomas with a mixed (*n* = 1), low‐grade endometrioid (*n* = 1), high‐grade endometrioid (*n* = 1), or poorly/undifferentiated (*n* = 4) carcinomas (Supp. Table S6B). All carcinomas that showed methylation of the promoter of *BRCA1* had a high‐grade serous histology (*n* = 9 patients). In contrast, high‐grade serous histology was observed in only three of the five carcinomas with somatic pathogenic mutations in *BRCA1* or *BRCA2*. Two patients with a somatic pathogenic mutation in *BRCA2* developed a carcinoma with either a mixed or poorly/undifferentiated histology (Table 2).

## Discussion

Reliable and sensitive analysis of the mutation status of *BRCA1* and *BRCA2* in FFPE OC samples is important, now that patients with germline and somatic pathogenic mutations in *BRCA1* and *BRCA2* are eligible for therapy with PARP inhibitors. Analysis of tumor DNA can be used as a prescreen for germline mutation analysis in blood only if germline mutations in tumor DNA can be assessed efficiently. Here, we show that reliable analysis of germline and somatic mutations is possible using a combination of smMIP‐based mutation detection and MLPA on DNA isolated from FFPE OCs.

Due to the high a priori risk of 10%–15% to carry a germline *BRCA1* or *BRCA2* mutation, all new OC patients are now eligible for germline DNA testing in many countries, including The Netherlands [Lancaster et al., 2015; Oncoline, 2015]. Our novel method enables reliable assessment of the tumor DNA mutation status of *BRCA1* and *BRCA2* in FFPE material derived from OCs. Performing such a *BRCA* tumor test on all newly diagnosed OC patients may be an efficient way to select all patients who may eventually benefit from treatment with PARP inhibitors and are simultaneously at high risk of carrying a pathogenic germline *BRCA* mutation, as approximately 75% of those that are tested positive for *BRCA* mutations in the tumor will have a germline mutation (i.e., hereditary predisposition; germline status of the mutation is confirmed in DNA derived from blood) [Cancer Genome Atlas Research, 2011]. This would limit genetic counselling procedures and concomitant distress to OC patients with a positive *BRCA* tumor test or a positive family history of ovarian cancer. This procedure would also be cost‐effective as it will reduce the number of germline tests and decreases double testing on both tumor and germline DNA. We are currently evaluating whether this altered diagnostic pathway starting with tumor DNA *BRCA* testing in newly diagnosed ovarian cancer as a prescreen for treatment and genetic counselling to initiate germline DNA testing on DNA derived from blood is feasible and is adopted as well as appreciated by both patients and professionals.

Most NGS approaches to determine the mutation status of *BRCA1* and *BRCA2* have been developed to sequence blood‐derived, high‐quality DNA and, consequently, can only be implemented in a routine diagnostic setting to screen for germline mutations in these genes [Feliubadalo et al., 2013; D'Argenio et al., 2015; Hirotsu et al., 2015; Strom et al., 2015; Trujillano et al., 2015]. Our smMIP‐based NGS approach provides double tiling of the ORF of *BRCA1* and *BRCA2* by targeting the plus and minus strand using independent overlapping smMIPs and enables the detection of unique reads by the introduction of a single‐molecule tag. The detection of unique reads enables the recognition of biased amplification of only a limited number of template molecules, which is commonly observed when a low amount of amplifiable input DNA is available (e.g., DNA derived from FFPE OCs). Formalin‐induced cytosine deamination artefacts, which frequently occur in DNA derived from FFPE samples [Chen et al., 2014], can be recognized due to the targeting of the plus and minus strand using independent overlapping smMIPs (i.e., double tiling). Our approach enables the reliable detection of mutations in *BRCA1* and *BRCA2* in DNA derived from FFPE material. Furthermore, since smMIP‐based NGS is a low‐cost and easily scalable method, extending this approach to sequence additional genes in FFPE samples is feasible [O'Roak et al., 2012; Kumar et al., 2014]. Since patients with defects in other homologous recombination genes may also benefit from PARP inhibitors, extending our smMIP design with probes targeting these genes could be considered [McCabe et al., 2006; Mateo et al., 2015]. This smMIP‐based analysis of *BRCA1* and *BRCA2* is a paradigm for reliable mutation analysis of complete ORFs or hotspot regions of other genes [Eijkelenboom et al., 2016].

If settings are used that filter out deamination artefacts, the amount of false‐positive calls using standard variant calling settings at a cut‐off value of 5% VAF is very low. In our analysis, no false‐positive calls were observed in 90% of the samples (96/107), whereas in nine samples only a single false‐positive variant was called and in two samples two and three false‐positive variants were called, respectively. All these calls could easily be recognized as false‐positive calls. Therefore, interpretation of sequencing results is very objective. The sensitivity of our smMIP‐based NGS approach was 97.8% based on standard variant calling of 810 *BRCA1* and *BRCA2* germline variants (100% was detected by visual inspection). Variants were missed either due to insufficient sequencing depth at three specific regions in samples with relatively low total coverage or due to loss of the allele containing the SNP, a phenomenon often observed in human cancers [Lengauer et al., 1998]. If germline mutations in *BRCA1* or *BRCA2* underlie the development of an OC, LOH will generally affect the allele without the causative germline mutation leading to an increase in mutant allele frequencies [Cancer Genome Atlas Research, 2011; Kanchi et al., 2014]. However, low frequent variant calls can reliably be detected using smMIP‐based NGS; we successfully identified somatic (passenger) variants with a low percentage of variant reads (e.g., 6% VAF). Noteworthy, the detection of low‐percentage variant calls can be easily improved by increasing the length of the molecular tag; a stretch of eight random nucleotides would enable the detection of 65,536, instead of 1,024, unique reads per smMIP [Hiatt et al., 2013]. Nevertheless, caution is required regarding the detection of germline variants in cancer samples with a high percentage of neoplastic cells (>90%), especially if mutation analysis is performed on tumor samples that are obtained after chemotherapy, as therapy may lead to reversion of the mutation [Norquist et al., 2011; Patch et al., 2015]. We have not yet optimized the detection of exon deletions and duplications that may inactivate *BRCA1* and *BRCA2* using our smMIP approach. For these aberrations, MLPA methods that are currently used in routine diagnostics of germline mutations can be applied.

In five of the 51 ovarian cancer patients without a germline *BRCA1* or *BRCA2* mutation included in this study, a somatic, pathogenic mutation affecting *BRCA1* or *BRCA2* was encountered. This confirms that these somatic mutations are relatively common in sporadic ovarian cancer patients [Cancer Genome Atlas Research, 2011; Kanchi et al., 2014]. In comparison with amplicon‐based target enrichment, our smMIP‐based approach has the advantage that the number of sequenced template molecules can be measured using the single‐molecule tag. This allows a proper risk estimation of the probability of false‐negative results given a chosen limit of detection [Eijkelenboom et al., 2016]. Our analyses were aimed at a minimal sequencing depth of 30 unique reads, which should be sufficient to detect >95% of variants present at a VAF of 20% or higher. On average, 97.4% of the ORF including 20 exon‐flanking nucleotides of *BRCA1* and *BRCA2* reached this sequencing depth, which was mostly much higher reflecting high complexity of our sequence libraries. Given the percentages of neoplastic cells in our samples was at least 40% in 90% of the tumor samples derived from sporadic patients, the chance that a somatic mutation was missed is low.

Acquired hypermethylation of the promoter of *BRCA1* in the OC was observed in 17% of the patients without a germline *BRCA* mutation. As described by others [Cancer Genome Atlas Research, 2011], hypermethylation was mutually exclusive with germline and somatic *BRCA1* and *BRCA2* mutations. In line with previous reports [Esteller et al., 2000], we noticed that LOH of *BRCA1* was observed in all OCs with methylation of the *BRCA1* promoter, suggesting that the hypermethylation is driving the tumourigenesis and will probably lead to homologous recombination‐deficient tumors. Therefore, patients who develop OCs with hypermethylation of the *BRCA1* promoter are predicted to benefit from PARP‐inhibitor treatments [Stefansson et al., 2012; Veeck et al., 2010]. A systematic evaluation of the effect of PARP inhibitors in this patient group seems justified.

It has been reported that the *BRCA1* or *BRCA2* gene is affected by a germline mutation, somatic mutation, or epigenetic silencing in approximately 33% of the high‐grade serous OCs [Cancer Genome Atlas Research, 2011]. Genomic aberrations affecting *BRCA1* and *BRCA2* are most common in high‐grade serous OCs, although they also occur in other subtypes [Hennessy et al., 2010; Alsop et al., 2012]. Our results support that the majority of the carriers of germline mutations in *BRCA1* and *BRCA2* develop (high‐grade) serous OCs [Boyd et al., 2000; Hennessy et al., 2010]; however, a significant subset (17%) of OCs derived from patients with germline mutations in *BRCA1* and *BRCA2* reveal a different histological phenotype. Therefore, we recommend that sequencing of *BRCA1* and *BRCA2* should be considered in all patients with OCs irrespective of their histological subtype.

In conclusion, a combined approach of smMIP‐based NGS, MLPA, and MS‐MLPA allows the reliable detection of both germline and somatic alterations affecting *BRCA1* and *BRCA2* in FFPE OCs (MLPA and MS‐MLPA were only performed for *BRCA1*). This approach enables the identification of (1) patients who may benefit from therapeutic treatments that are based on the mutation status of *BRCA1* and *BRCA2* (e.g., PARP‐inhibitors) and (2) those at high risk of a pathogenic germline mutation in *BRCA1* and *BRCA2* (i.e., hereditary predisposition). Therefore, our approach will further improve clinical treatment and hereditary cancer risk assessment of ovarian cancer patients.

## Supporting information

Disclaimer: Supplementary materials have been peer‐reviewed but not copyedited.

Supp. Fig. S1. **A**: Correlation between the quality of input DNA and the achieved sequencing depth. Sequencing depth of BRCA1 and BRCA2 was poor in samples not fulfilling DNA quality settings (red dots, N=16) compared to samples fulfilling these criteria (black dots, N=111) based on the percentage of the ORF (including ‐20 and +20 intronic regions) with a sequencing depth of at least 30x. DNA quality was assessed based on amplifiability (i.e. DNA fragments of 115bp and 216bp could successfully be amplified in a control PCR) and concentration (i.e. >2.5ng/μl) (see materials and methods). **B**: Correlation between the total number of mapped reads and sequencing depth per targeted basepair. Insufficient coverage of the open reading frame of BRCA1 and BRCA2, especially observed for input samples of poor DNA quality (see Supp. Figure S1a), strongly correlates with a low number of total mapped reads, implicating an consistent distribution of mapped reads over the entire ORF. **C**: Average number of unique reads per base pair. Y‐axis: average number of unique reads. Bars on the x‐axis represent the nucleotides located in the exons of BRCA1 and BRCA2, including the canonical splice sites. White and black blocks represent alternating exons. **D**: Total number of tagged reads and unique reads mapped to the open‐reading frame of BRCA1 and BRCA2. A median of 2,085,329 and 2,898,671 tagged reads mapping to the open‐reading frame of BRCA1 and BRCA2 were obtained per sample (n=107 ovarian cancer samples). These tagged reads were grouped based on their barcodes (five random nucleotides, see materials and methods) to create (a median of) 61,107 and 87,155 unique reads mapping to the open‐reading frame of BRCA1 and BRCA2. **E**: Total number of mapped reads per FFPE ovarian carcinoma sample. On average, 105,949 reads were mapped to the ORF of BRCA1 or BRCA2. In five samples with a low number of mapped reads (red bars), 13 previously identified SNPs were not properly called and could only be confirmed by visual inspection of the data. Arrows indicate the number of false positive variant calls per sample. For details, see main text.
Supp. Fig. S2. Correlation between the number and percentage of variant reads and true/false variants called in BRCA1 and BRCA2. **A**: Validated germline (green dots) and somatic (black squares) insertions and deletions have a higher number of variant reads and percentage of variant reads compared to false calls (red crosses). **B**: Similar to indels, germline SNPs (green dots) and somatic SNVs (black squares) have higher numbers and percentages of variant reads compared to deamination artefacts (grey dots) and false positive SNV calls (red crosses). Indels: Insertions and deletions. SNP: single nucleotide polymorphism. SNV: single nucleotide variant. X‐axis: number of unique variant reads. Y‐axis: percentage of variant reads.
Supp. Fig. S3. CNV analysis based on smMIP‐based NGS data to detect exon 22 deletions in BRCA1. The number of unique reads per smMIP was first corrected for the total number of unique reads per sample. Subsequently, these numbers were adjusted based on the average number of reads per smMIP in all samples (i.e. average number of unique reads per smMIP in 107 samples is 1). Next, the average number of reads per exon was determined per sample (average of multiple smMIPs targeting the same exon). Orange and pink dots reflect the average number of reads per exon for two exon 22 deletion carriers, P005 and P090, respectively. Black dots reflect the average number of reads per exon for 105 non‐deletion carriers. Gray arrows point towards the strongly decreased numbers of unique reads targeting exon 22 in P005 and P090. Note: four tumours derived from three patients who carried a germline deletion encompassing exon 22 were sequenced (see main text), but the sequencing depth/number of mapped reads was too low to analyze for two samples.
Supp. Fig. S4. Distribution of LOH informative SNPs located on chromosome 13 and 17. Most informative SNPs (5≤95% VAF in at least one sample), depicted by red dots, are located at the genomic loci of BRCA1 and BRCA2. For details, see Supp. Table S3.

Supp. Table S1. Sequences of the BRCA‐targeting single molecule molecular inversion probes. This supplementary table is provided in the supplementary Excel file.
Supp. Table S2. Variants called in 107 FFPE OC samples that are not present in our in‐house database of germline BRCA1 and BRCA2 variants. This supplementary table is provided in the supplementary Excel file.
Supp. Table S3. Rate of concordance of BRCA1 methylation status in tumour samples at diagnosis and later in the therapeutic process.
Supp. Table S4. LOH analysis of 107 FFPE OC samples based on heterozygous SNVs at chromosome 17 (BRCA1) and chromosome 13 (BRCA2). This supplementary table is provided in the supplementary Excel file.
Supp. Table S5. LOH analysis based on the A‐allele frequency of available informative SNPs at the BRCA loci.
Supp. Table S6. **A**: Pathological review of the ovarian tumours derived from germline BRCA mutation carriers. **B**: Pathological review of the ovarian tumours derived from sporadic patients
