# Supplementary material for: Novel BRCA1 and BRCA2 Tumor Test as Basis for Treatment Decisions and Referral for Genetic Counselling of Patients with Ovarian Carcinomas
Source: Hum Mutat. 2016 Nov 9;38(2):226–35. doi: 10.1002/humu.23137 (PMC5248611; doi:10.1002/humu.23137)
Supplement: Supplementary file 1 — Supp. Fig. S1. A: Correlation between the quality of input DNA and the achieved sequencing depth. Sequencing depth of BRCA1 and BRCA2 was poor in samples not fulfilling DNA quality settings (red dots, N=16) compared to samples fulfilling these criteria (black dots, N=111) based on the percentage of the ORF (including ‐20 and +20 intronic regions) with a sequencing depth of at least 30x. DNA quality was assessed based on amplifiability (i.e. DNA fragments of 115bp and 216bp could successfully be amplified in a control PCR) and concentration (i.e. >2.5ng/μl) (see materials and methods). B: Correlation between the total number of mapped reads and sequencing depth per targeted basepair. Insufficient coverage of the open reading frame of BRCA1 and BRCA2, especially observed for input samples of poor DNA quality (see Supp. Figure S1a), strongly correlates with a low number of total mapped reads, implicating an consistent distribution of mapped reads over the entire ORF. C: Average number of unique reads per base pair. Y‐axis: average number of unique reads. Bars on the x‐axis represent the nucleotides located in the exons of BRCA1 and BRCA2, including the canonical splice sites. White and black blocks represent alternating exons. D: Total number of tagged reads and unique reads mapped to the open‐reading frame of BRCA1 and BRCA2. A median of 2,085,329 and 2,898,671 tagged reads mapping to the open‐reading frame of BRCA1 and BRCA2 were obtained per sample (n=107 ovarian cancer samples). These tagged reads were grouped based on their barcodes (five random nucleotides, see materials and methods) to create (a median of) 61,107 and 87,155 unique reads mapping to the open‐reading frame of BRCA1 and BRCA2. E: Total number of mapped reads per FFPE ovarian carcinoma sample. On average, 105,949 reads were mapped to the ORF of BRCA1 or BRCA2. In five samples with a low number of mapped reads (red bars), 13 previously identified SNPs were not properly called and could only be confirm [file HUMU-38-226-s001.doc]

**Novel tumour DNA *BRCA1* and *BRCA2* test as basis for treatment decisions and referral for genetic counselling of patients with ovarian carcinomas**

Robbert D.A. Weren^1^, Arjen R. Mensenkamp^1^, Michiel Simons^2^, Astrid Eijkelenboom^2^, Aisha S. Sie^1^, Hicham Ouchene^1^, Monique van Asseldonk^2^, Encarna B. Gomez-Garcia^3^, Marinus J. Blok^3^, Joanne A. de Hullu^4^, Marcel R. Nelen^1^, Alexander Hoischen^1^, Johan Bulten^2^, Bastiaan B.J. Tops^2^, Nicoline Hoogerbrugge^1^ and Marjolijn J.L. Ligtenberg^1,2^.

| **Online Supporting Information** | **Page** |
| --- | --- |
| **Supplementary Figure S1a. Correlation between the quality of input DNA and the achieved sequencing depth.** | 2 |
| **Supplementary Figure S1b. Correlation between the total number of mapped reads and sequencing depth per targeted basepair.** | 2 |
| **Supplementary Figure S1c. Total number of tagged reads and unique reads mapped to the open-reading frame of *BRCA1* and *BRCA2*.** | 3 |
| **Supplementary Figure S1d. Number of unique reads/smMIPs mapping to/targeting the plus or minus strand of the open-reading frame of *BRCA1* and *BRCA2*.** | 4 |
| **Supplementary Figure S1e. Total number of mapped reads per FFPE ovarian carcinoma sample.** | 5 |
| **Supplementary Figure S2. Correlation between the number and percentage of variant reads and true/false variants called in *BRCA1* and *BRCA2*.** | 6 |
| **Supplementary Figure S3. CNV analysis based on smMIP-based NGS data to detect exon 22 deletions in *BRCA1*.** | 7 |
| **Supplementary Figure S4. Distribution of LOH informative SNPs located on chromosome 13 and 17.** | 8 |
| **Supplementary Table S1. Sequences of the BRCA-targeting single molecule molecular inversion probes.** | 9 |
| **Supplementary Table S2. Variants called in 107 FFPE OC samples that are not present in our in-house database of germline *BRCA1* and *BRCA2* variants.** | 9 |
| **Supplementary Table S3. Rate of concordance of *BRCA1* methylation status in tumour samples at diagnosis and later in the therapeutic process.** | 9 |
| **Supplementary Table S4. LOH analysis of 107 FFPE OC samples based on heterozygous SNVs at chromosome 17 (*BRCA1*) and chromosome 13 (*BRCA2*).** | 9 |
| **Supplementary Table S5. LOH analysis based on the A-allele frequency of available informative SNPs at the *BRCA* loci.** | 10 |
| **Supplementary Table S6a. Pathological review of the ovarian tumours derived from germline *BRCA* mutation carriers.** | 11 |
| **Supplementary Table S6b. Pathological review of the ovarian tumours derived from sporadic patients** | 12 |
| **Supplementary Materials and Methods** | 13 |

**Supplementary Information**


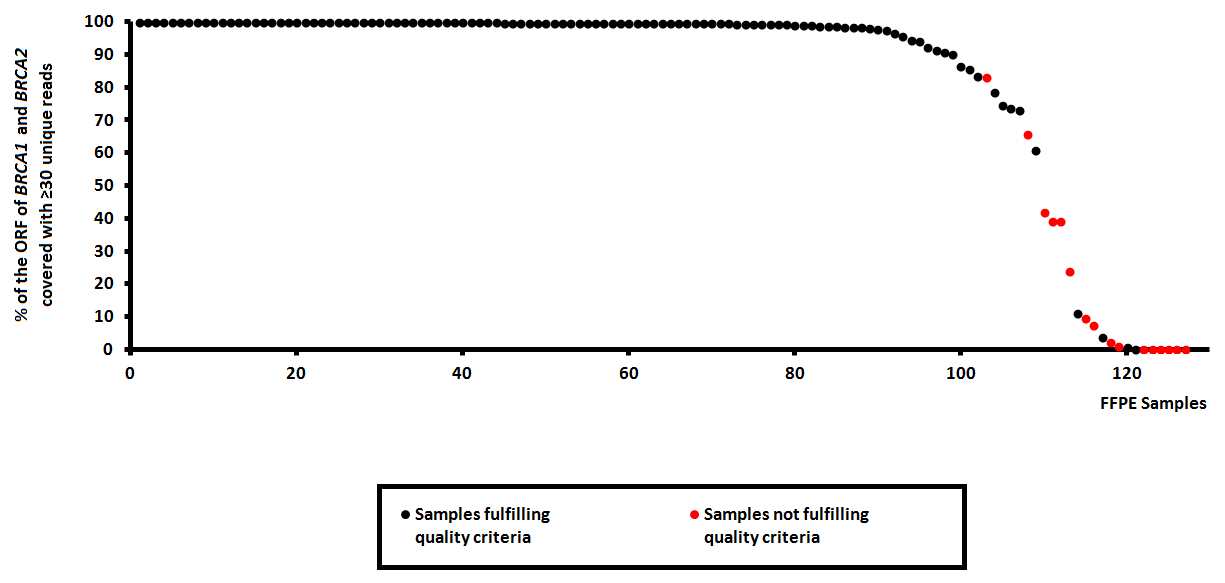


**Supplementary Figure S1a. Correlation between the quality of input DNA and the achieved sequencing depth.** Sequencing depth of *BRCA1* and *BRCA2* was poor in samples not fulfilling DNA quality settings (red dots, *N*=16) compared to samples fulfilling these criteria (black dots, *N*=111) based on the percentage of the ORF (including -20 and +20 intronic regions) with a sequencing depth of at least 30x. DNA quality was assessed based on amplifiability (i.e. DNA fragments of 115bp and 216bp could successfully be amplified in a control PCR) and concentration (i.e. >2.5ng/µl) (see materials and methods).


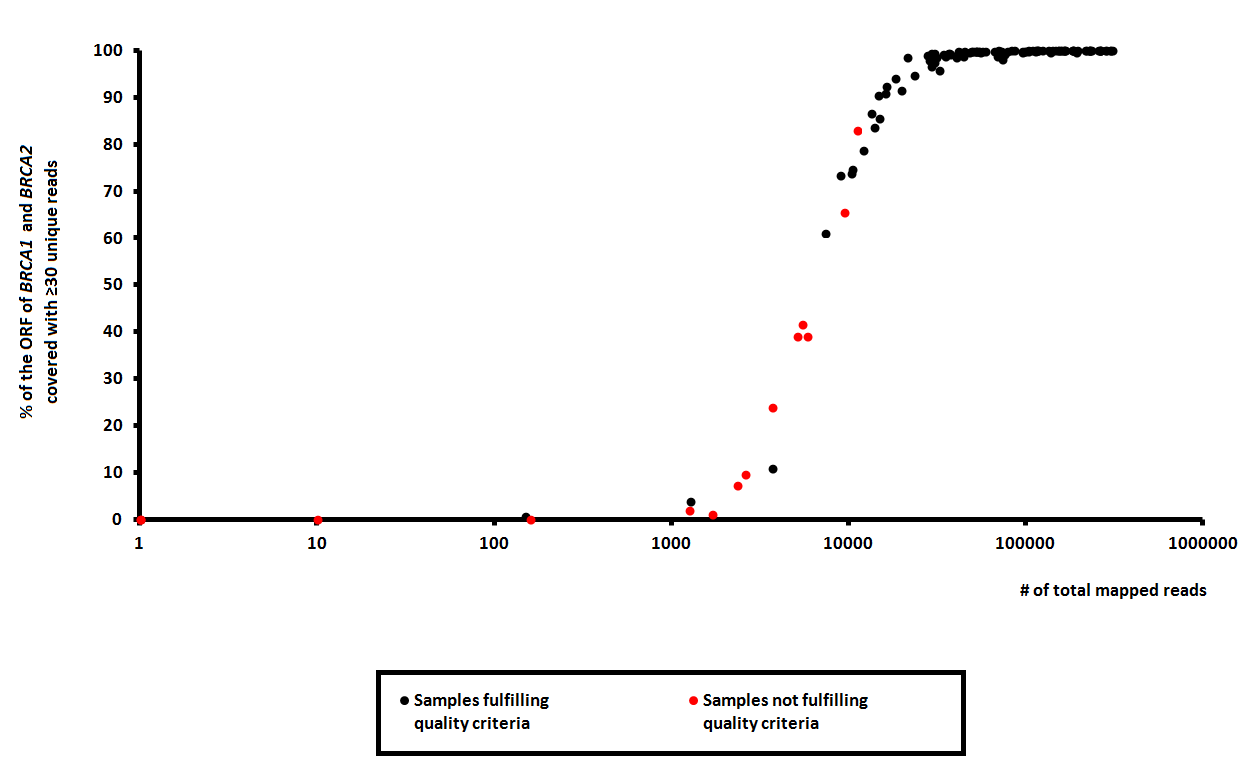


**Supplementary Figure S1b. Correlation between the total number of mapped reads and sequencing depth per targeted basepair.** Insufficient coverage of the open reading frame of *BRCA1* and *BRCA2*, especially observed for input samples of poor DNA quality (see **Supp. Figure S1a**), strongly correlates with a low number of total mapped reads, implicating an consistent distribution of mapped reads over the entire ORF.


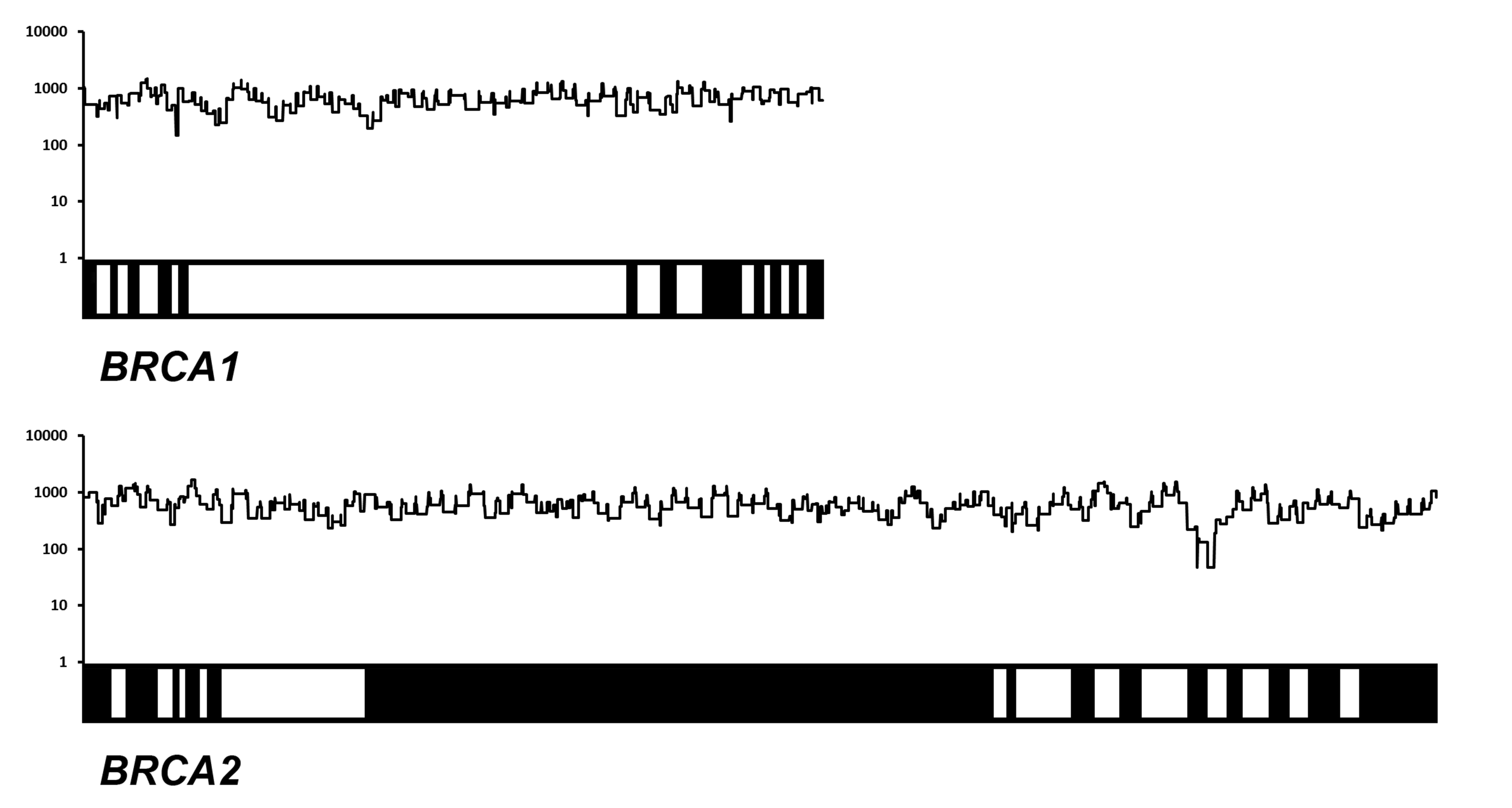


**Supplementary Figure S1c. Average number of unique reads per base pair.** Y-axis: average number of unique reads. Bars on the x-axis represent the nucleotides located in the exons of *BRCA1* and *BRCA2*, including the canonical splice sites. White and black blocks represent alternating exons.


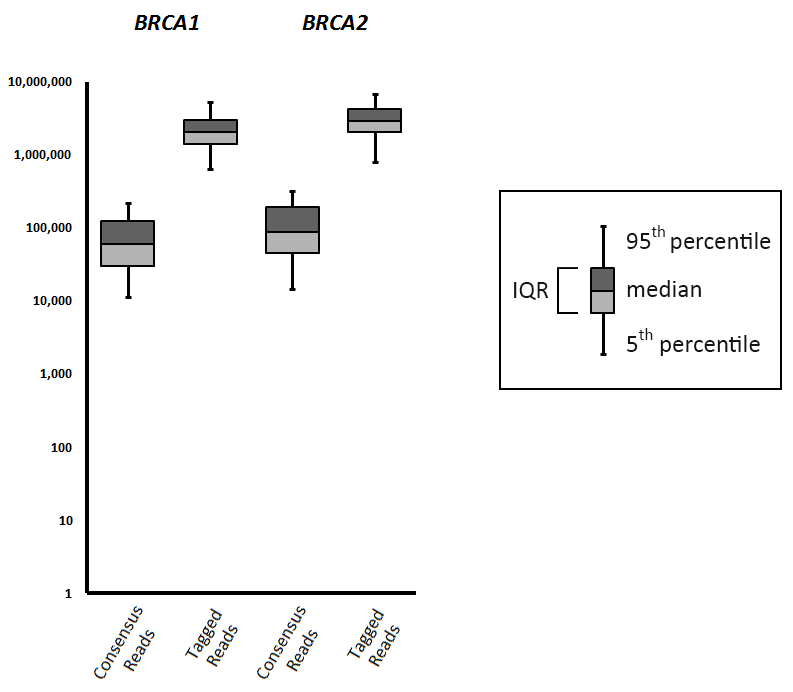


**Supplementary Figure S1d. Total number of tagged reads and unique reads mapped to the open-reading frame of *BRCA1* and *BRCA2*.** A median of 2,085,329 and 2,898,671 tagged reads mapping to the open-reading frame of *BRCA1* and *BRCA2* were obtained per sample (*n*=107 ovarian cancer samples). These tagged reads were grouped based on their barcodes (five random nucleotides, see materials and methods) to create (a median of) 61,107 and 87,155 unique reads mapping to the open-reading frame of *BRCA1* and *BRCA2*.

**
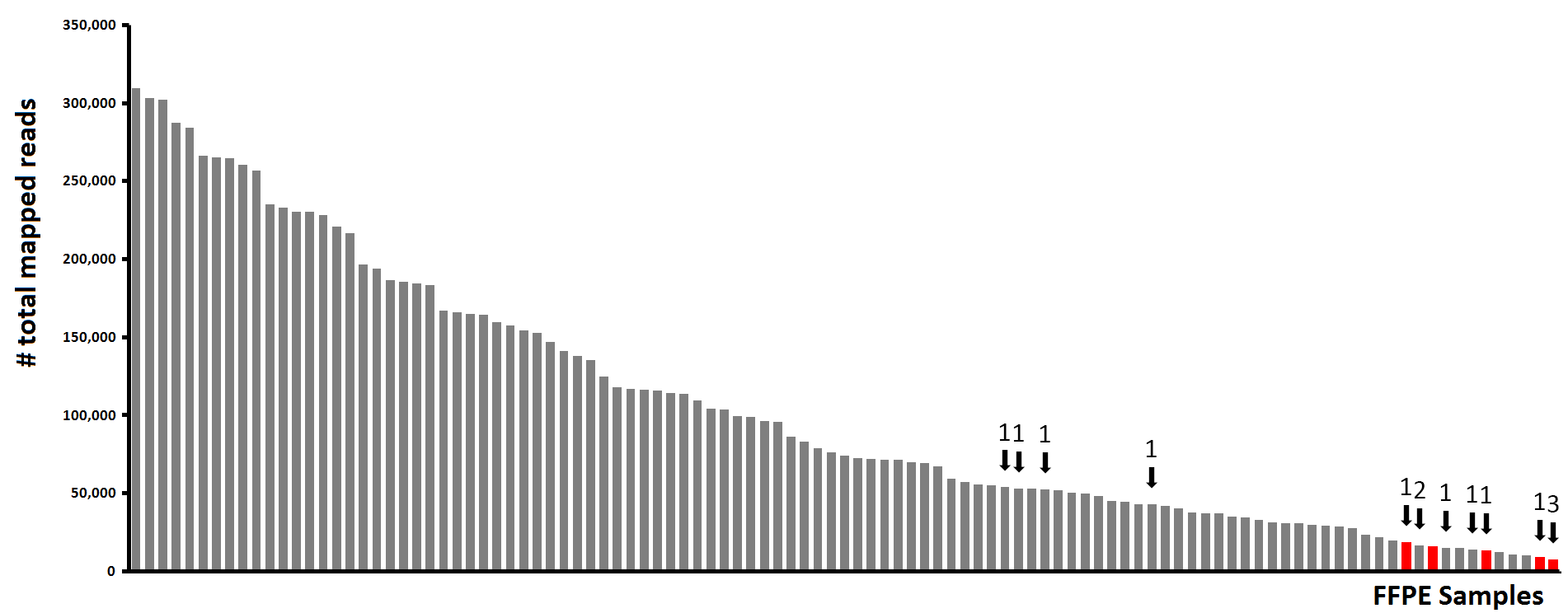
**

**Supplementary Figure S1e. Total number of mapped reads per FFPE ovarian carcinoma sample.** On average, 105,949 reads were mapped to the ORF of *BRCA1* or *BRCA2*. In five samples with a low number of mapped reads (red bars), 13 previously identified SNPs were not properly called and could only be confirmed by visual inspection of the data. Arrows indicate the number of false positive variant calls per sample. For details, see main text.

**
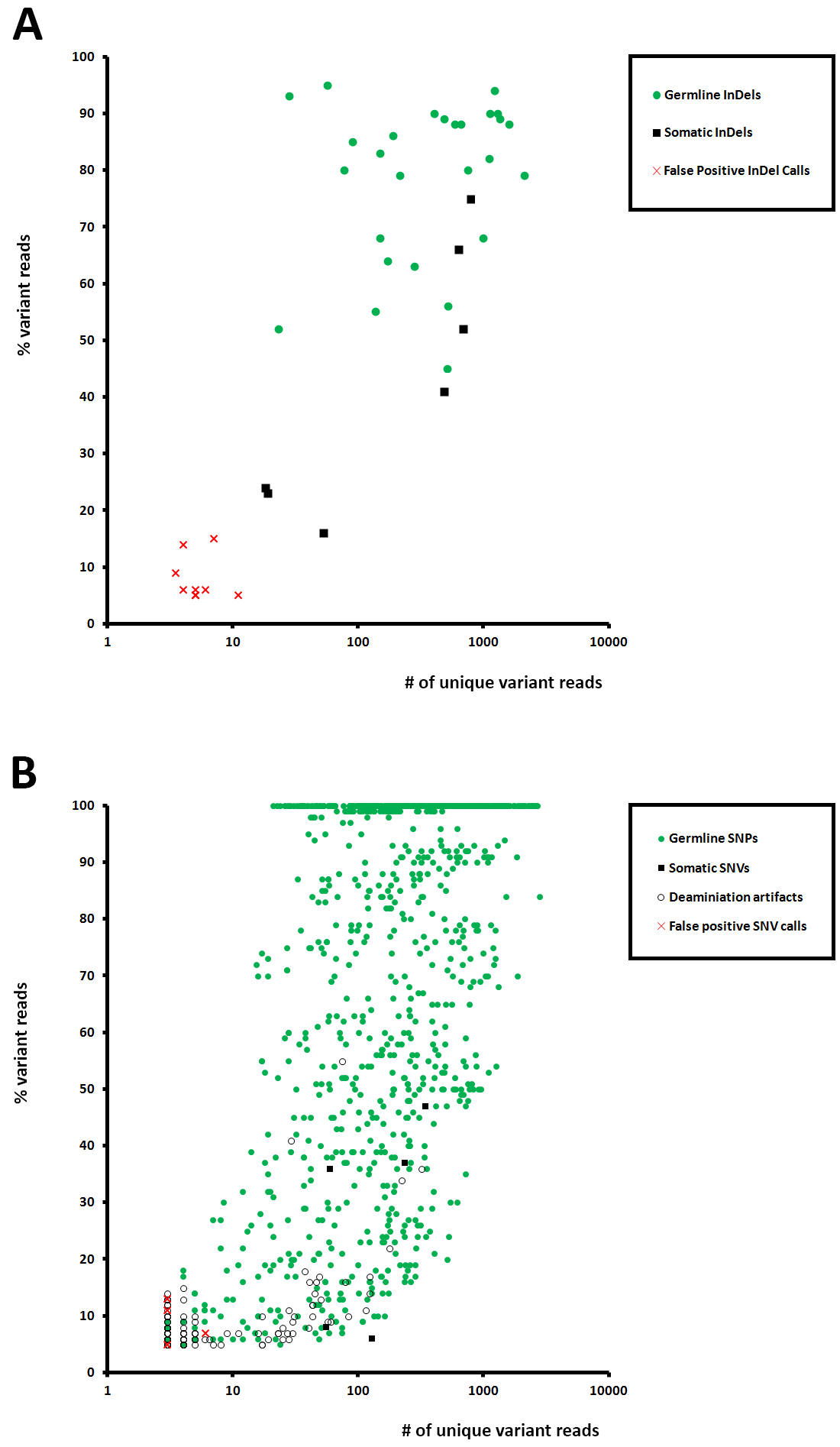
**

**Supplementary Figure S2. Correlation between the number and percentage of variant reads and true/false variants called in *BRCA1* and *BRCA2*.** A) Validated germline (green dots) and somatic (black squares) insertions and deletions have a higher number of variant reads and percentage of variant reads compared to false calls (red crosses). B) Similar to indels, germline SNPs (green dots) and somatic SNVs (black squares) have higher numbers and percentages of variant reads compared to deamination artefacts (grey dots) and false positive SNV calls (red crosses). Indels: Insertions and deletions. SNP: single nucleotide polymorphism. SNV: single nucleotide variant. X-axis: number of unique variant reads. Y-axis: percentage of variant reads.


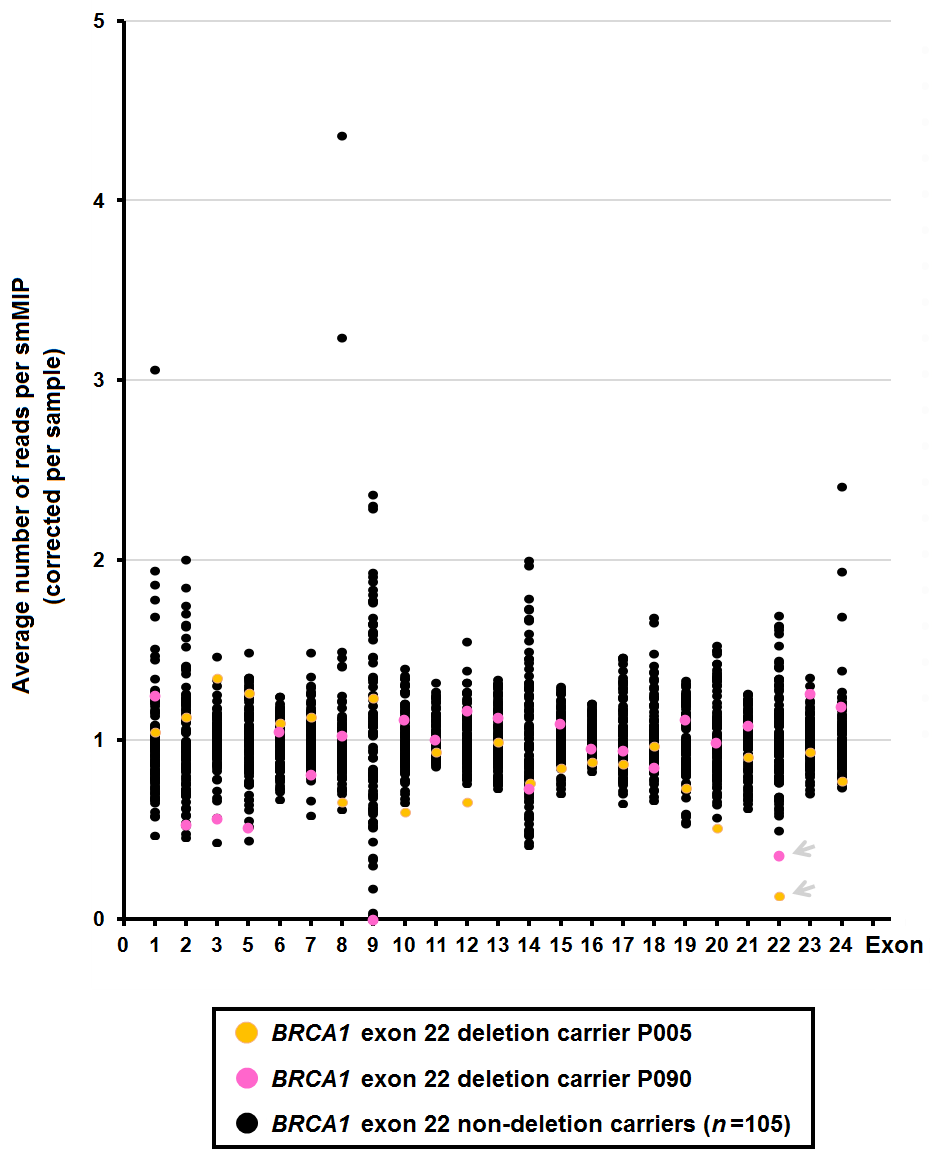


**Supplementary Figure S3. CNV analysis based on smMIP-based NGS data to detect exon 22 deletions in *BRCA1*.** The number of unique reads per smMIP was first corrected for the total number of unique reads per sample. Subsequently, these numbers were adjusted based on the average number of reads per smMIP in all samples (i.e. average number of unique reads per smMIP in 107 samples is 1). Next, the average number of reads per exon was determined per sample (average of multiple smMIPs targeting the same exon). Orange and pink dots reflect the average number of reads per exon for two exon 22 deletion carriers, P005 and P090, respectively. Black dots reflect the average number of reads per exon for 105 non-deletion carriers. Gray arrows point towards the strongly decreased numbers of unique reads targeting exon 22 in P005 and P090. Note: four tumours derived from three patients who carried a germline deletion encompassing exon 22 were sequenced (see main text), but the sequencing depth/number of mapped reads was too low to analyze for two samples.

**
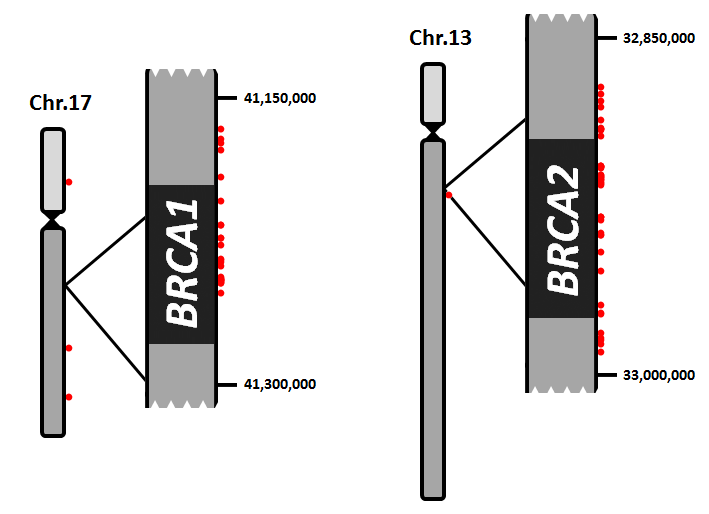
**

**Supplementary Figure S4. Distribution of LOH informative SNPs located on chromosome 13 and 17.** Most informative SNPs (5≤95% VAF in at least one sample), depicted by red dots, are located at the genomic loci of *BRCA1* and *BRCA2*. For details, see **Supp. Table S3**.

**Supplementary Table S1. Sequences of the *BRCA*-targeting single molecule molecular inversion probes.** This supplementary table is provided in the supplementary Excel file.

**Supplementary Table S2. Variants called in 107 FFPE OC samples that are not present in our in-house database of germline *BRCA1* and *BRCA2* variants.** This supplementary table is provided in the supplementary Excel file.

**Supplementary Table S3. Rate of concordance of *BRCA1* methylation status in tumour samples at diagnosis and later in the therapeutic process.**

| **Patient** | **Mutation status** | **Methylation of the BRCA1 promoter**  **(Diagnostic)** | **Methylation of the BRCA1 promoter**  **(Post-chemotherapy)** |
| --- | --- | --- | --- |
| **P005** | *BRCA1* ; Germline | No | No |
| **P016** | *BRCA1* ; Germline | No | No |
| **P035** | *BRCA1* ; Germline | No | No |
| **P038** | *BRCA1* ; Germline | No | No |
| **P040** | *BRCA1* ; Germline | No | No |
| **P057** | *BRCA1* ; Germline | No | No |
| **P070** | *BRCA1* ; Germline | No | No |
| **P078** | *BRCA1* ; Germline | No | No |
| **P028** | *BRCA2* ; Germline | No | No |
| **P073** | *BRCA2* ; Germline | No | No |
| **P086** | *BRCA2* ; Germline | No | No |
| **P047** | *BRCA1* ; Somatic | No | No |
| **P050** | *BRCA2* ; Somatic | No | No |
| **P002** | Sporadic | No | No |
| **P008** | Sporadic | No | No |
| **P011** | Sporadic | No | No |
| **P012** | Sporadic | No | No |
| **P015** | Sporadic | No | No |
| **P033** | Sporadic | No | No |
| **P037** | Sporadic | **Yes** | **Yes** |
| **P059** | Sporadic | No | No |
| **P065** | Sporadic | No | No |
| **P083** | Sporadic | **Yes** | **Yes** |
| **P087** | Sporadic | **Yes** | **Yes** |

**Supplementary Table S4. LOH analysis of 107 FFPE OC samples based on heterozygous SNVs at chromosome 17 (*BRCA1*) and chromosome 13 (*BRCA2*).** This supplementary table is provided in the supplementary Excel file.

**Supplementary Table S5. LOH analysis based on the A-allele frequency of available informative SNPs at the *BRCA* loci.**

| **Mutation status sample** | **Locus** | ***#samples*** | ***% of samples with LOH*** | **median (%)** | **min (%)** | **max (%)** | **# samples without informative SNPs** |
| --- | --- | --- | --- | --- | --- | --- | --- |
| ***BRCA1* mutation; germline** | *BRCA1* | 33 | 100 | 83 | 68 | 92 | 1 |
| ***BRCA1* mutation; somatic** | *BRCA1* | 4 | 100 | 61 | 60 | 73 | 0 |
| ***BRCA1;* promoter methylation** | *BRCA1* | 10 | 100 | 81 | 62 | 94 | 1 |
| ***BRCA2* mutation; germline** | *BRCA1* | 16 | 73 | 65 | 52 | 91 | 1 |
| **sporadic, no *BRCA1* mutation/methylation** | *BRCA1* | 44 | 80 | 78 | 52 | 95 | 4 |
|  |  |  |  |  |  |  |  |
| ***BRCA2* mutation; germline** | *BRCA2* | 16 | 73 | 67 | 52 | 92 | 1 |
| ***BRCA2* mutation; somatic** | *BRCA2* | 4 | 100 | 81 | 73 | 89 | 0 |
| ***BRCA1* mutation; germline** | *BRCA2* | 33 | 56 | 65 | 51 | 95 | 1 |
| **sporadic; no *BRCA2* mutation** | *BRCA2* | 54 | 46 | 60 | 51 | 94 | 2 |

**Supplementary Table S6a. Pathological review of the ovarian tumours derived from germline *BRCA*** mutation carriers.

| **Patient ID** | **Germline mutation** | **Carcinoma phenotype** |
| --- | --- | --- |
| **P001** | *BRCA1* | Poorly / undifferentiated |
| **P005** | *BRCA1* | High grade serous |
| **P007** | *BRCA1* | High grade serous |
| **P016** | *BRCA1* | High grade serous |
| **P022** | *BRCA1* | High grade serous |
| **P025** | *BRCA1* | High grade serous |
| **P029** | *BRCA1* | High grade serous |
| **P035** | *BRCA1* | High grade serous |
| **P036** | *BRCA1* | High grade serous |
| **P038** | *BRCA1* | High grade serous |
| **P039** | *BRCA1* | High grade serous |
| **P040** | *BRCA1* | High grade serous |
| **P046** | *BRCA1* | High grade serous |
| **P048** | *BRCA1* | High grade serous |
| **P057** | *BRCA1* | High grade serous |
| **P066** | *BRCA1* | High grade serous |
| **P070** | *BRCA1* | High grade serous |
| **P076** | *BRCA1* | Mixed carcinoma |
| **P077** | *BRCA1* | High grade serous |
| **P078** | *BRCA1* | Clear cell |
| **P079** | *BRCA1* | Poorly / undifferentiated |
| **P084** | *BRCA1* | High grade serous |
| **P085** | *BRCA1* | Mixed carcinoma |
| **P088** | *BRCA1* | High grade serous |
| **P090** | *BRCA1* | High grade serous |
| **P093** | *BRCA1* | High grade serous |
| **P094** | *BRCA1* | High grade serous |
| **P096** | *BRCA1* | High grade endometrioid |
| **P006** | *BRCA2* | High grade serous |
| **P021** | *BRCA2* | High grade serous |
| **P028** | *BRCA2* | High grade serous |
| **P034** | *BRCA2* | High grade serous |
| **P044** | *BRCA2* | High grade serous |
| **P067** | *BRCA2* | High grade serous |
| **P068** | *BRCA2* | High grade serous |
| **P069** | *BRCA2* | High grade serous |
| **P071** | *BRCA2* | High grade serous |
| **P072** | *BRCA2* | High grade serous |
| **P073** | *BRCA2* | High grade serous |
| **P074** | *BRCA2* | High grade serous |
| **P075** | *BRCA2* | Poorly / undifferentiated |
| **P086** | *BRCA2* | High grade serous |

**Supplementary Table S6b. Pathological review of the ovarian tumours** derived from sporadic patients

| **Patient ID** | ***BRCA1* methylation^1^** | **Somatic mutation^2^** | **Carcinoma phenotype** |
| --- | --- | --- | --- |
| **P002** | No | No | High grade serous^3^ |
| **P003** | No | No | High grade serous |
| **P004** | Yes | No | High grade serous |
| **P008** | No | No | Low grade serous^3^ |
| **P009** | No | No | High grade serous |
| **P010** | No | No | High grade endometrioid |
| **P011** | No | No | High grade serous |
| **P012** | No | No | High grade serous |
| **P013** | No | No | High grade serous |
| **P014** | No | No | Low grade serous |
| **P015** | No | No | High grade serous |
| **P017** | No | No | High grade serous |
| **P018** | Yes | No | High grade serous |
| **P019** | No | No | Poorly / undifferentiated |
| **P020** | No | No | Low grade serous |
| **P023** | Yes | No | High grade serous |
| **P024** | No | No | High grade serous |
| **P026** | No | No | High grade serous |
| **P027** | No | No | High grade serous |
| **P030** | No | No | High grade serous |
| **P031** | No | No | High grade serous |
| **P032** | No | No | High grade serous |
| **P033** | No | No | High grade serous |
| **P037** | Yes | No | High grade serous |
| **P041** | No | No | High grade serous |
| **P042** | No | No | High grade serous |
| **P043** | No | No | High grade serous |
| **P045** | No | No | High grade serous |
| **P047** | No | Yes; *BRCA1* | High grade serous |
| **P049** | No | No | High grade serous |
| **P050** | No | Yes; *BRCA2* | Mixed carcinoma |
| **P051** | No | No | High grade serous |
| **P052** | No | No | High grade serous |
| **P053** | No | No | High grade serous |
| **P054** | No | No | Poorly / undifferentiated |
| **P055** | No | No | High grade serous |
| **P056** | Yes | No | High grade serous |
| **P058** | No | No | High grade serous |
| **P059** | No | No | High grade serous |
| **P060** | No | No | High grade serous |
| **P061** | No | Yes; *BRCA1* | High grade serous |
| **P062** | No | Yes; *BRCA2* | Poorly / undifferentiated |
| **P063** | No | No | Low grade endometrioid |
| **P064** | No | No | High grade serous |
| **P065** | No | No | High grade serous |
| **P080** | No | No | High grade serous |
| **P081** | Yes | No | High grade serous |
| **P082** | No | No | High grade serous |
| **P083** | Yes | No | High grade serous |
| **P087** | Yes | No | High grade serous |
| **P089** | Yes | No | High grade serous |
| **P091** | No | Yes; *BRCA2* | High grade serous |
| **P092** | No | No | Poorly / undifferentiated |
| **P095** | No | No | High grade serous |

^1^ Hypermethylation of the *BRCA1* promoter.

^2^ Somatic pathogenic mutation detected, see **Table 2**.

^3^ Two tumours derived from these patients are considered low- and high grade serous, respectively, possibly reflecting tumour dedifferentiation.

**Supplementary Materials and Methods**

**FFPE-derived DNA amplifiability**

To establish the quality of FFPE derived DNA, amplifiability of the DNA was assessed by PCR-based amplification of DNA fragments of 115bp and 216bp. To amplify these DNA fragments, the following primers were used:

| **Primer name** | **Primer Sequence** |
| --- | --- |
| *KRAS*_Fw | TGTAAAACGACGGCCAGTAGGCCTGCTGAAAATGACTG |
| *FSHR*_Fw | CTACCCTGCACAAAGACAGTG |
| *KRAS*_Rv | CAGGAAACAGCTATGACCTGGATCATATTCGTCCACAAAA |
| *FSHR*_Rv | GTGTACGTCATGTCAAATCCTCTGC |

The subsequent PCR reaction, using Amplitaq Gold 360 master mix (ThermoFisher), was performed using the following cycle conditions: 95°C, 10 min – 38x [95°C, 30 sec - 58°C, 30 sec - 72°C, 1 min] - 72°C, 7 min - 20°C, ∞.

***In-silico* predictions somatic single nucleotide variants**

*In-silico* predictions were performed according to the guidelines previously published (Vihinen, 2013). To estimate the possible pathogenecity of four somatic missense variants in *BRCA2* (**Table 2**), *in-silico* predictions were performed using the Alamut Visual software package, version 2.7.1; April 2015 (Interactive Biosoftware, Rouen, France) (accessed 05/2016). *In-silico* predictions were based on PhyloP (Pollard, et al., 2010), SIFT (Kumar, et al., 2009) and AlignGVGD (Mathe, et al., 2006; Tavtigian, et al., 2006).
